# Diversity of oat varieties in eliciting the early inflammatory events in celiac disease

**DOI:** 10.1007/s00394-013-0617-4

**Published:** 2013-11-19

**Authors:** Marco Silano, Elena Penas Pozo, Francesca Uberti, Sara Manferdelli, Tamara Del Pinto, Cristina Felli, Andrea Budelli, Olimpia Vincentini, Patrizia Restani

**Affiliations:** 1 Unit of Human Nutrition and Health, Department of Veterinary Public Health and Food Safety, Istituto Superiore di Sanità , Viale Regina Elena 299, 00161 Rome, Italy; 2 University of Milan, Milan, Italy; 3 Infant & Nutrition, Heinz Italia S.p.A., Latina, Italy; 4 Department of Therapeutic Research and Medicines Evaluation, Istituto Superiore di Sanità, Rome, Italy

**Keywords:** Celiac disease, Gluten-free diet, Oats, Avenins

## Abstract

**Purpose:**

Celiac disease (CD) is an autoimmune enteropathy, triggered by dietary gluten. The only treatment is a strict gluten-free diet. Oats are included in the list of gluten-free ingredients by European Regulation, but the safety of oats in CD is still a matter of debate. The present study examined the capability of different oat cultivars of activating the gliadin-induced transglutaminase-2 (TG2)-dependent events in some in vitro models of CD. In addition, we compared this capability with the electrophoresis pattern of peptic–tryptic digests of the proteins of the oat cultivars.

**Methods:**

K562(S) cells agglutination, transepithelial electrical resistance of T84-cell monolayers, intracellular levels of TG2 and phosphorylated form of protein 42–44 in T84 cells were the early gliadin-dependent events studied.

**Results:**

The results showed that the Nave oat cultivar elicited these events, whereas Irina and Potenza varieties did not. The ability of a cultivar to activate the above-described events was associated with the electrophoretic pattern of oat proteins and their reactivity to anti-gliadin antibodies.

**Conclusion:**

We found significant differences among oat cultivars in eliciting the TG2-mediated events of CD inflammation. Therefore, the safety of an oat cultivar in CD might be screened in vitro by means of biochemical and biological assays, before starting a clinical trial to definitely assess its safety.

## Introduction

Celiac disease (CD) is a permanent autoimmune enteropathy, triggered in genetically predisposed individuals by dietary gluten [1, 2]. Gluten is a storage-protein fraction of some cereals, such as wheat, rye and barley. Wheat gluten encompasses two fractions: the alcohol-soluble gliadins (monomeric) and the acid acetic-soluble glutenins (polymeric, whose subunits are linked to each others by disulfide bonds). Gliadins are further classified in α- , β- , γ- and ϖ-gliadins according to their electrophoretic mobility, glutenins in high and low molecular weight fraction. Both gliadins and glutenins are toxic in CD. α-gliadin is the main protein of wheat gluten [3].

In recent years, a growing body of evidence has shown that the gliadin-dependent inflammation in celiac duodenal mucosa is the result of a complex interaction of both innate and adaptive immunity. In fact, the presentation of gliadin epitopes by means of DQ2/8+ antigen-presenting cells to mucosal T cells is not sufficient to explain all the aspects of the duodenal mucosa inflammation in CD [4]. It has been recently described that the activation of the innate immunity by the “toxic” peptides, such as p31–43, sets the tone of the mucosal response to gliadin, and it is required for the downstream recognition of gliadin-specific CD4+ T cells [5]. The response to p31–43 in celiac inflammation encompasses some very precocious transglutaminase 2 (TG2)-dependent key events that occur at the surface of intestinal epithelial cells, including increased expression and activity of the enzyme TG2 itself, cytoskeleton rearrangement and protein phosphorylation [6, 7].

The prevalence of CD is estimated to be roughly 1 % worldwide, and it is the most frequent food intolerance [8]. At present, the only treatment for this condition is the life-long complete withdrawal of gluten from the diet [9, 10]. Strict adherence to a gluten-free diet (GFD) is required to control the symptoms of CD and to prevent the autoimmune and neoplastic complications associated with this condition [11]. However, full compliance with GFD heavily affects dietary choice and the quality of life. Although the quality of gluten-free foods has significantly improved in the last decades, some problems still remain partially unresolved, in particular the lower technological performances of gluten-free cereals [12].

On these bases, the inclusion of oats in GFD could be of great value. Oats are a good source of fiber and in particular β-glucans, which are healthy compounds that reduce serum LDL cholesterol and the glycemic index of foodstuffs [13, 14]. Moreover, the inclusion of oats in GFD can offer a wider choice for celiac patients. Although oats are included among the gluten-free ingredients by European Commission Regulation 41/2009 [15], the safety of oats in CD is still a matter of debate. Some clinical trials have concluded that oats are well tolerated by CD patients on GFD [16–19], but earlier studies found that some patients consuming oats as part of GFD suffered an intestinal inflammation similar to that in untreated celiac patients [20–22].

These contradictory results might be explained by the fact that the variety of oats used in the challenges was not taken into account. Oats include many varieties, naked and husked seeds, containing various amino acid sequences [23] and showing different immunoreactivities to anti-gliadin polyclonal antibodies [24]. We have previously reported that the prolamin avenins (the proteins corresponding to wheat gliadin) from different oat cultivars display different immunostimulatory activities on celiac lymphocytes from peripheral blood [25]. Comino et al. [26] have confirmed that the immunogenicity of oats on celiac T lymphocytes differs for different cultivars and showed a direct correlation between the immunogenic ability of the oat cultivars and the concentration of the immunostimulatory gliadin peptide 33mer in their protein fraction.

However, the activation of T cells by immunogenic p33mer is a very late event in the inflammatory cascade of CD; a grain to be considered safe in CD should also not trigger the very early TG2-dependent events of epithelial activation [5].

We have therefore studied different oat cultivars in in vitro and ex vivo models of celiac epithelial activation and compared the capacity to activate this pathway with the oat protein profile.

## Materials and methods

### Cereal samples

Three naked cultivars of *Avena sativa* L. (cv. Irina, cv. Potenza e cv. Nave) were included in the study. Oat cultivars Irina and Potenza were provided by Heinz Italia S.p.A. (Latina, Italy). Seeds were finely ground by a coffee mill exclusively used for gluten-free ingredients. Rice (*Oryza sativa* L.) and wheat (*Triticum aestivum* L.) flours were used as negative and positive controls, respectively. Gluten contamination in the samples of oats and rice was assessed by RIDASCREEN^®^ Gliadin ELISA kit (R-Biopharm AG, Darmstadt, Germany).

### Peptic–tryptic digestion of cereal proteins

The proteolysis of oats, wheat and rice flours was performed by sequential digestion with purified pepsin and trypsin. Five grams of each cereal flour was suspended in 50 mL of 0.1 N HCl containing 10 mg of pepsin from porcine gastric mucosa (EC 3.4.23.1, Merck, Damstadt, Germany) and was incubated at pH 2.0 and 37 °C for 3 min, 10 min or 2 h. Then, the pH of the samples was adjusted to 8.0, with 0.2 N NaOH and 10 mg of trypsin from bovine pancreas (EC 3.4.21.4, Sigma, Milan, Italy) was added and allowed to act for 2 or 4 h. The digestion was stopped by adjusting at pH 7 with 0.1 N HCl. All digestions were performed at least in duplicate. Incubations without enzymes under the same conditions were performed to obtain undigested samples (Time 0).

For SDS-polyacrylamide gel electrophoresis (PAGE) analysis, 1 mL of each digestion solution was diluted (1:1; v/v) with sample buffer containing 0.125 M Tris–HCl pH 6.8, 3.75 % glycerol, 1 % SDS and 5 % β-mercaptoethanol. For the in vitro studies on cells, an aliquot (5 mL) of each digestion solution was freeze dried (freeze-dryer Edwards Modulyo, UK).

Oats and rice digests were tested to be gluten contamination-free with the ELISA kit-containing antibodies versus R5-peptide (R-Biopharm, Darmstadt, Germany). All the digests were tested endotoxin-free using the Pyrotell Limulus amebocyte Lysate assay (Cape Cod Inc., Falmouth MA, USA).

### SDS-PAGE

The electrophoresis profile of oats, rice and wheat flours as well as that of their corresponding digestion products was analyzed by SDS-PAGE under reducing conditions in a gel having the following composition:

- *Gradient running gel* 9–19 % acrylamide, 0.08–0.17 % bis-acrylamide, 0.36 M Tris–HCl buffer pH 8.8, 35 % glycerol, 0.1 % SDS, 0.02 % ammonium persulfate and 0.15 % *N*,*N*,*N*′,*N*′-tetramethylenediamine (TEMED).
- *Stacking gel* 3.5 % acrylamide, 0.09 % bis-acrylamide, 0.125 M Tris–HCl buffer pH 6.8, 0.1 % SDS, 0.02 % ammonium persulfate and 0.15 % TEMED.
- *Running buffer* 25 mM Tris–HCl pH 8.8, 0.19 M glycine and 0.1 % SDS (w/v).

Prestained molecular weight marker solution (broad range, Bio-Rad) contained myosin (192.8 kDa), β-galactosidase (117.9 kDa), bovine serum albumin (99.3 kDa), ovalbumin (54.1 kDa), carbonic anhydrase (37.8 kDa), soybean trypsin inhibitor (29.5 kDa), lysozyme (20.2 kDa) and aprotinin (7.4 kDa). After the electrophoretic run (90 V at room temperature for approximately 6 h), gels were dyed with Coomassie Brilliant Blue G-250. All materials and instruments were purchased from Bio-Rad (Hercules, CA, USA).

### Immunoblotting

The immunoreactivity in samples was tested using immunoblotting and a commercial antibody specifically developed against wheat gliadins. After SDS-PAGE, proteins were transferred to a PVDF membrane (Millipore, Billerica, MA) by Western blotting in a Trans-blot Electrophoretic Transfer Cell (Bio-Rad). The membranes were blocked with 1 % gelatin and washed three times with 0.25 % gelatin solution (150 mM NaCl, 5 mM Tris and 0.05 % Triton X) to prevent nonspecific adsorption of the immunological reagents. The membrane was then immersed in 10 mL of 0.25 % gelatin solution containing 10 μL of a rabbit anti-wheat gliadin polyclonal antibody (Sigma Aldrich, Italy), and antigen–IgG complexes were detected using 10 μL of mouse anti-rabbit IgG monoclonal antibodies labeled with alkaline phosphatase (Sigma Aldrich Italia). After incubation in the bromochloroindolyl phosphate-nitroblue tetrazolium (BCIP/NBT) solution, a black-purple precipitate developed at the site of the enzyme binding. The developing solution contained 15 % bromochloroindolyl phosphate and 30 % nitroblue tetrazolium in alkaline phosphatase buffer (100 mM Tris, 100 mM sodium chloride and 5 mM magnesium chloride, pH 9.5).

### Cell lines

Human leukemic K562(S) and human colon adenocarcinoma T84 cell lines were obtained from American Type Culture Collection (commercialized in Italy by LGC Standard, Milan) and cultured as previously described [27].

### K562(S) cell agglutination test

The K562(S) cell agglutination test was performed as previously described [27]. Briefly, K562(S) cells were harvested by centrifugation and washed twice with Ca^2+^ and Mg^2+^-free PBS (GIBCO, Carlsbad, CA, USA) and resuspended at a concentration of 10^8^ cells/mL in the same PBS. Twenty-five μL of cell suspension was added to each well of a 96-well microtiter plate containing the PT digest of the cereals (7 mg/mL). The final total volume was 100 μL. The cell suspension was incubated at room temperature (RT) for 30 min. The agglutinating activity of the different digests was measured using a 96 plate reader equipped with a stirrer (Bio-Rad, Hercules, CA, USA). The cell suspension turbidity was read at 600 nm (OD 600 nm) under continuous stirring at time 0 and after 30 min. The difference in readings between that at T0 and T30 × 100 was calculated as a velocity of agglutination (CV).

### Measurement of transepithelial electrical resistance across T84 cell monolayer

T84 cells were seeded on polycarbonate inserts (0.45 mm pore diameter, 0.9 cm^2^ area; (BD Falcon, Franklin Lakes, NJ, USA) and left to grow to full confluence for at least 19 days, in order to establish a cell monolayer. The formation of the cell monolayer was ensured by measuring a transepithelial electrical resistance (TEER) value of at least 800 Ω ms/mm^2^, using a Millicell ERS device (Millipore, Bedford, MA, USA). The digestion products from different cereals studied in this paper were added (1 mg/mL at 37 °C) to the culture medium of T84 cells for 3 h. The impact of the different digests on monolayer permeability was expressed as the difference in the TEER value measured after 3-h incubation with the digest, and the TEER value measured just before the addition of the digest to the cell culture (ΔΩ ms/mm^2^).

### Western blotting of p42–44 and TG2 protein in T84 cells

T84 cells were seeded in 6-well plates and challenged after 5 days, at the pre-confluence stage, with the PT digests of cereals (1 mg/mL) for 3 h for the determination of p42–44 expression and 24 h for the determination of TG2 expression. Independently, whole cell extracts were washed twice in ice-cold PBS, resuspended in 150 mM NaCl, 1 % Triton X-100 1 % (Sigma, St Louis, MO, USA) and a mixture of protease inhibitors (1:50) (Sigma) and incubated on ice for 20 min and then centrifuged for 5 min at 4 °C. The supernatant was stored at −70 °C as whole cell protein lysate. SDS–PAGE was carried out on 4 % stacking and 7.5 % resolving gel (Bio-Rad, Hercules, CA, USA). Equal amounts of protein (50 mg) were loaded in each lane with loading buffer containing 0.1 Tris (pH 6.8), 20 % glycerol, 10 % mercaptoethanol, 4 % SDS and 0.2 % Bromophenol Blue (Bio-Rad). Samples were heated at 100 °C for 5 min before gel loading. After electrophoresis, the proteins were transferred to a PVDF membrane (Bio-Rad). Membranes were blocked for 1 h with 5 % nonfat milk in TBS (100 mM NaCl, 5 mM KCl, 100 mM Tris–HCl, pH 7.4 and 0.05 % Tween 20, Bio-Rad) and incubated overnight with phosphorylated form of p42–44 (mouse Ab, used at dilution 1:1,000, Cell Signaling, Danvers, MA, USA) or TG2 antibody (mouse Ab, used at dilution 1:100, clone CUB 7402, Abcam, Cambridge, MA, USA) and then washed three times for 5 min in TBST. Secondary antibody (goat-antimouse, conjugated to horse-radish peroxidase HRP) (Bio-Rad) was diluted 1:3,000 in the blocking solution, added to membranes for 1 h at RT and then washed three times in TBS for 5 min. Proteins on membranes were revealed by the chemiluminescence detection kit (Bio-Rad), according to manufacturer’s instructions. Intensities of protein bands on blots were measured using the Bio-Rad ChemiDoc densitometer. Membranes were stripped and reprobed with β-actin antibody (Abcam) diluted 1:400 to verify equal loading of proteins. The density of the blots was analyzed with a densitometric software (Bio-Rad), which quantifies the intensity of the bands. The figures so obtained were normalized for the corresponding actin intensity and expressed in a graph.

### Patients

Duodenal biopsies were performed for diagnostic purposes in three untreated celiac patients. The histological and serological profiles of these patients are described in Table 1. Written informed consent was obtained from parents of the children involved, and the study was approved by the Ethics Committee of Istituto Superiore di Sanità. One specimen from each patient was used for diagnosis; the other samples were cultured in vitro as follows.

**Table 1:** Clinical characteristics of the three celiac children enrolled in the study

Patient ID	Age (years)	Gender	Anti-TG2 IgA titer (U/mL)	EMA	Histological lesions of duodenal mucosa (according to Marsh–Oberhuber classification)
1	7.5	M	87	+++	3c
2	10.8	F	58	++	3b
3	8.1	F	71	+++	3c

### In vitro organ culture of biopsy specimens from celiac patients

Duodenal specimens were put on a metallic grid in a Petri’s dish and partially immersed in RPMI supplemented with FCS (10 %), penicillin/streptomycin, HEPES and nonessential amino acids and glutamine. The specimens were exposed to the PT digest (1 mg/mL) of different cereals for 3 h and then embedded in optimal cutting temperature (Bioptica, Milano, Italy). Tissue sections of 5 μm obtained in the cryostat were fixed in acetone to methanol 1:1 for 20 min at 4 °C and incubated with a 1 % BSA for 20 min to prevent nonspecific antibody binding. The sections were then incubated with TG2 antibody (CUB7402 Abcam; 1:1000) for 1 h at RT and then with AlexaFluor 660 secondary antibody (1:500, Life Technologies) for 60 min at RT. The images were acquired with a fluorescence microscope.

### Statistical analysis

Cells treated with PT digests of oats or wheat flour were compared with cells treated with PT digests of rice flour. All experiments were performed in triplicate. Data distribution was analyzed and statistical differences were evaluated using Wilcoxon test and SPSS 12 software. A *P* value of <0.05 was considered significant.

## Results

### Oats protein profiles

**Fig. 1:**
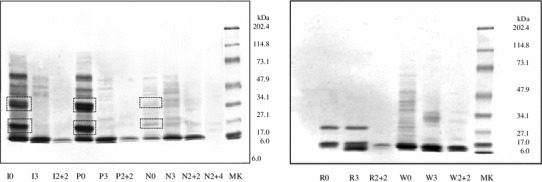
SDS-PAGE pattern of three different oat cultivars, rice and wheat flours digested for different times of digestion with pepsin or pepsin/trypsin. The *outlined boxes* include the bands encompassing the avenins fractions. I = oats var. Irina; P = oats var. Potenza; N = oats var. Nave; R = rice; W = bread wheat; MK = molecular weight standard solution; 0 = undigested samples; 3 = digested with pepsin for 3 min; 2 + 2 = digested for 2 h with pepsin + 2 h with trypsin; and 2 + 4 = digested for 2 h with pepsin + 4 h with trypsin

The protein profiles of oats, rice and wheat samples at some of the different times and types of digestion performed in this study (nondigested, pepsin for 3 min, pepsin for 2 h and then trypsin for 2 h, pepsin for 2 h and then trypsin for 4 h) obtained with SDS-PAGE are illustrated in Fig. 1. The patterns of undigested Irina, Potenza and Nave oat cultivars showed a mixture of proteins including oat prolamins (outlined boxes in the figure). Pepsin digestion for 3 min brought about significant proteolysis in both Irina and Potenza oats: the number and intensity of protein bands decreased progressively during pepsin digestion for 10 min and 2 h (results not shown) and almost disappeared after the combined action of pepsin (2 h) and trypsin (both 2 and 4 h). Oats cv. Nave presented a different protein distribution and a general lower abundance. Pepsin digestion for 3 min of Nave oats caused little proteolysis and even after the combined action of pepsin (2 h) plus trypsin (2 h), a significant amount of avenins was still present; it took trypsin digestion for 4 h to remove them completely. Rice flour presented three main protein bands with molecular weights ranging from 6 to 30 kDa. Digestion of rice with pepsin for 3 and 10 min (data not shown) had little effect in its protein pattern, but digestion with trypsin for 2 h virtually removed all the bands; whereas, the lowest molecular weight proteins of wheat digests resisted to trypsin attack.

**Fig. 2:**
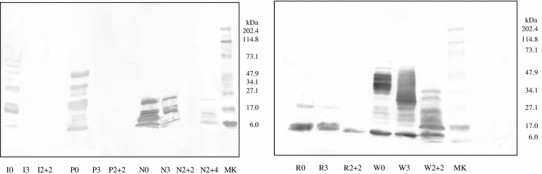
Immunoblotting of the different digestion products illustrated in Fig. 1, obtained after incubation with rabbit anti-gliadin polyclonal antibodies. I = oats var. Irina; P = oats var. Potenza; N = oats var. Nave; R = rice; W = bread wheat; MK = molecular weight standard solution; 0 = undigested samples; 3 = digested with pepsin for 3 min; 2 + 2 = digested for 2 h with pepsin + 2 h with trypsin; and 2 + 4 = digested for 2 h with pepsin + 4 h with trypsin

Figure 2 shows the results of immunoblotting obtained after incubation of the cereals samples, illustrated in Fig. 1, with polyclonal anti-gliadin antibodies. Undigested oats cv. Irina and Potenza showed a very low reactivity, which completely disappeared at digestion time as short as 3 min with pepsin. The Nave cultivar presented high immunoreactivity at time 0 decreasing after 2 h of pepsin and trypsin and a weak increase after 4 h of trypsin, which could be due to the production of reactive fragments after prolonged attack. Like the oats, undigested rice showed a weak immunoreactivity to the anti-gliadin antibody (Fig. 2, right), but in contrast, this binding survived peptic and tryptic digestions. As expected, wheat samples showed strong immunochemical reactivity throughout all digestion periods.

### Agglutination of K562(S) cells

**Fig. 3:**
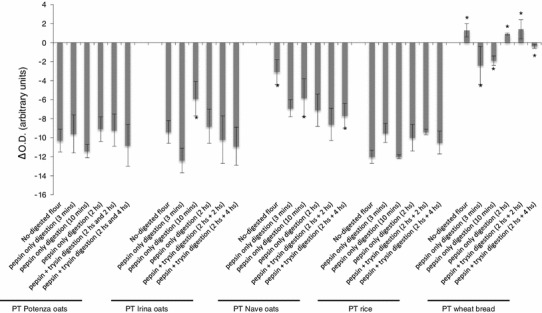
Measurement of the agglutination velocity of K562(S) cells incubated with the flour PT digest of the mentioned cereals, progressively digested. K562(S) agglutination is a TG2-dependent mechanism induced only by cereal toxic in CD. The agglutination velocity is expressed as the difference in reading at 600 nm with a plate reader of cell suspension turbidity at time 0 and time 30 min after the incubation with the digests. Higher the difference is, higher the agglutination velocity is. Bread wheat only resulted to induce a fast K562(S) cell agglutination, regardless the time of digestion, whereas the incubation of the cells with flour rice digests do not result in cell agglutination. Results are expressed as mean ± SD of three different experiments, each performed in triplicate. *P < 0.05. Statistical analysis performed by Wilcoxon test versus cells incubated with the corresponding rice PT digest (negative control)

We have previously shown [27] that the agglutination of myelogenic leukemia cells K562(S) by toxic gliadin peptides relies upon the activity of cellular TG2, an enzyme involved in the very precocious events triggered by these peptides at the cell surface of duodenal epithelial cells. Briefly, the Ca^2+^-dependent TG2 activation triggered by p31–43 in K562 cells leads to cytoskeleton rearrangement, which is a key event in cell agglutination. This cell line therefore offers a useful and rapid tool for evaluating the ability of a cereal to trigger the epithelial activation in CD. We incubated oat cultivar digests with K562(S) cells for as little as 30 min before reading the agglutination time (Fig. 3). The negative control (rice flour) did not induce cell agglutination for any time of PT digestion, whereas wheat flour, used as positive control, triggered a massive agglutination of the K562(S) cells regardless of the time of PT digestion. The exposure of K562(S) cells to the PT digests of both oat cultivars Irina and Potenza did not result in cell agglutination, whereas some of the PT digests from oat cultivar Nave did agglutinate the K562(S) cells, though with a lower agglutination velocity than wheat flour. This result confirms the toxicity previously described for Nave oats in some in vitro models of CD inflammation [25].

### Permeability of T84 cell monolayer

Gliadin-dependent early events, similar to those responsible for K562(S) cells agglutination, also occur at the surface of the gliadin-sensitive cell line T84 [6]. The cytoskeleton rearrangement induced by p31–43 in these cells causes an increased permeability of the cell monolayer, when T84 cells are grown on a polycarbonate filter in a bidimensional cell culture system [27]. We screened the effect on the TEER of a T84 cell monolayer after a 3 h incubation with the PT digests.

**Fig. 4:**
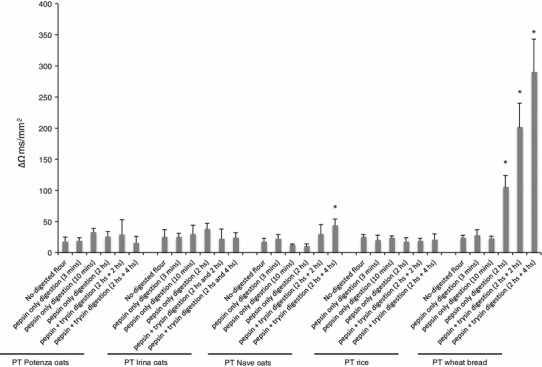
Variation in the TEER in T84 cells grown on a filter of polycarbonate. Results are expressed as the difference in the TEER value (ΔΩ ms/mm^2)^ measured after 3-h of incubation with the digests and the TEER value measured just before the addition of the digests to the cell culture. The positive variation in the TEER is a cell surface-associated event induced by cereal toxic in celiac disease. Bread wheat only resulted to induce a significant variation in the TEER. Results are expressed as mean ± SD of three different experiments, each performed in triplicate. *P < 0.05. Statistical analysis performed by Wilcoxon test versus cells incubated with the corresponding rice PT digest (negative control)

As shown in Fig. 4, the incubation of the bidimensional cell culture system with all the wheat PT digests resulted in a significant decrease in the TEER compared with oats PT digests. Regarding the oats, only the Nave oats digested with pepsin and trypsin for the longest time affected the TEER; no effect was noticed for PT digests of Irina and Potenza oats.

At this point of the experimental procedure, it was clear to us that wheat flour was capable to trigger the celiac epithelial activation regardless the type and the time of digestion. In the meantime, all PT digests of rice flour were found not toxic. Concerning the oats, all the PT digests of Irina and Potenza cultivars were shown to be not toxic, whereas only Nave digested with pepsin for 2 h and trypsin for 4 h elicited the cell surface events. So, we decided to use only this type of digestion, which fully mimics the human gastro-intestinal digestion, for the following more complex and time-demanding experiments of this study. The toxicity observed in this in vitro test is fully in agreement with proteolysis pattern as observed in SDS-PAGE and immunoreactivity illustrated in Fig. 2.

### TG2 and p42–44 expression in T84 cells

P31–43 accumulates into the lysosome, impacting on the TG2-degradation machinery. The increased intracellular levels of TG2 led to a PPAR-γ downregulation, which is a key event in the derangement of the intracellular environment of celiac intestinal epithelial cells [7]. The phosphorylation of cellular protein 42–44 has been demonstrated to be a good index of early cereal toxicity in CD [6, 28].

**Fig. 5:**
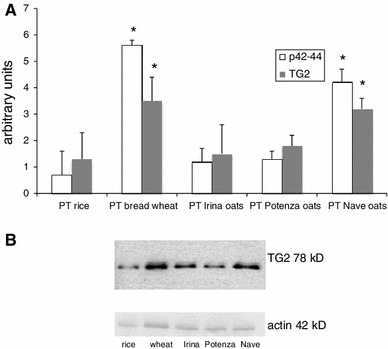
**a** Expression of gliadin-dependent epithelial pro-inflammatory molecules p42–44 and TG2 in T84 cells exposed in vitro for three and 24 h, respectively, to PT digests of the cereals indicated, as resulted to Western blotting analysis. The density of the blots was quantified with a densometric software and was normalized for actin expression. Results are expressed as mean ± SD of three different experiments, each performed in triplicate. *P < 0.05. Statistical analysis performed by Wilcoxon test versus PT rice-treated group. **b** The figure represents the result of an experiment out of the three WB performed

Therefore, we analyzed the expression of both TG2 and phosphorylated p42–44 in T84 cells treated with the PT digests of oats flours. As expected, the incubation of cells with wheat dramatically over-expressed TG2 and p42–44 compared with cells treated with rice-flour digest. The incubation of T84 cells with Irina or Potenza oats did not exert any effect on the cellular expression of these two molecules, whereas the digest of Nave oats increased the levels of TG2 and p42–44 in a similar way to wheat (Fig. 5).

**Fig. 6:**
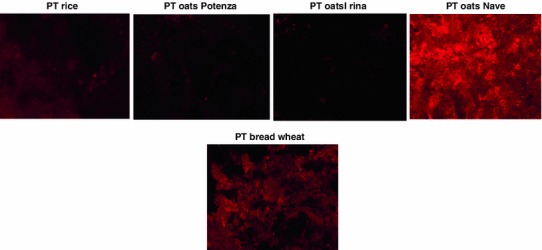
Expression of TG2 in celiac duodenal mucosa specimens challenged in vitro with the peptic–tryptic digest (PT) of the indicated flour (1 mg/mL) for 24 h. The increase expression of TG2 in celiac small bowel mucosa is a very precocious event of gliadin toxicity in celiac inflammation. The incubation of mucosa specimens with flour PT digests of Potenza and Irina oat cultivars failed to induce TG2 expression in the mucosa specimens, as the digest of rice flour. On the contrary, PT digest of oat cultivar Nave induced a marked TG2 expression in celiac mucosa specimens, in a similar manner of PT digest of the bread wheat. Magnification ×40

To confirm the results obtained with Western blotting, we performed the TG2-immunofluorescence staining on sections of celiac duodenal specimens exposed in vitro to oats PT digests. TG2 expression increased in celiac mucosa when exposed overnight to PT digests of wheat and Nave oats compared with samples exposed to rice digest; on the contrary, the TG2 expression in mucosa sections incubated with Irina and Potenza oats resulted comparable with that of specimens incubated with rice flour (Fig. 6).

## Discussion

In this study, we have shown that there is a significant difference among oat cultivars in eliciting the very precocious events occurring at the cell surface and triggering the downstream mucosa inflammation in CD. Furthermore, we found an association between the toxicity of oat cultivar digests and the electrophoresis pattern and the reactivity to anti-gliadin polyclonal antibodies.

Fragments derived from the gastro-intestinal digestion of gliadin activate specifically the innate or the adaptive immune system in celiac mucosa. The former are termed “toxic” peptides, the latter “immunodominant” peptides. Among the toxic sequences, p31–43 is the best known [5, 29], but several other peptides are able to act as inductors of this stage of the immunity in celiac mucosa [30]. Six gliadin epitopes have been found so far and all of them are included within the sequence of a 33-amino acid long peptide, which is resistant to the proteolysis by human gastro-intestinal enzymes [31]. The exposure of duodenal mucosa to p31–43 is required not only to predispose the T lymphocytes to the late adaptive immune response, but also to induce some very early inflammatory events, occurring at the surface of epithelial cells [32]. On the contrary, the exposure of the celiac duodenal mucosa to any of gliadin immunomodulatory epitopes alone does not exert any effect. Recent studies have shown that both phases of immune response to gliadin are governed by the activity of the TG2 [7]. This enzyme plays a role so essential in CD pathogenesis that its down-regulation is currently under study as an alternative therapy to GFD [10]. All the events investigated in this in vitro study are TG2 dependent. Particularly, Irina and Potenza oats, differently from Nave oats, do not impact the TG2-mediated cytoskeleton rearrangement in K562(S) and T84 cell lines and the TG2 expression in T84 cells and celiac mucosa. Although it is always difficult to compare different studies, we confirm the results obtained by Maglio et al. [33] that showed that Potenza oats completely fail to activate the innate immune response in human intestinal epithelial cell line Caco-2 and celiac duodenal mucosa. These authors, however, reported that Potenza oats in vitro increased the number of T lymphocytes in specimens of celiac duodenal mucosa. As discussed above, this event occurs late in the downstream inflammatory cascade pathogenetic in CD and requires the trigger of the mucosal innate immunity. Therefore, it does not necessarily indicate that this oats is toxic in CD.

Our data give further support to the conclusions by Comino et al. [26], who described that some oat cultivars have no immunogenic activity on celiac peripheral lymphocytes. Although we tested different oat varieties, the present paper completes and integrates these findings, describing the oats impact on the very early TG2-dependent inflammatory CD events, whereas the paper by Comino et al. investigated downstream events in the inflammatory cascade of CD.

The toxicity observed in in vitro tests was fully in agreement with proteolysis pattern as observed in SDS-PAGE and immunoreactivity versus polyclonal anti-gliadin antibodies. We have used polyclonal antibodies to identify the potential toxic fragments within avenins sequences rather than searching the presence of p31–43, since this sequence is only one among several gliadin toxic peptides, whose exact number and sequences are still unknown. Moreover, potential oats toxic peptides could have significant differences in amino acid sequences. Oats is a cereal that includes numerous cultivars; they widely differ between protein content and avenins composition, which can be responsible for the different affinities observed between prolamins and anti-gliadin antibodies [34]. The weak immunoreactivity to anti-gliadin antibodies showed by rice digests might be due to a nonspecific binding of the polyclonal antibodies (reacting not only to toxic sequences) to rice proteins, whereas the R5 antibody is a monoclonal antibody that recognizes very specifically the gluten toxic sequence QQPFP [35].

To conclude, the inclusion of oats in a GFD might be valuable for the nutritional and health benefits of this cereal. Although the definitive proof whether a specific oat cultivar is suitable for CD patients may come from randomized double-blind placebo-controlled clinical trial, it might be a useful preliminary in vitro screening of the cultivar toxicity or safety by means of biochemical and biologocial assays.
